# Earth-like aqueous debris-flow activity on Mars at high orbital obliquity in the last million years

**DOI:** 10.1038/ncomms8543

**Published:** 2015-06-23

**Authors:** T. de Haas, E. Hauber, S. J. Conway, H. van Steijn, A. Johnsson, M. G. Kleinhans

**Affiliations:** 1 Faculty of Geosciences, Universiteit Utrecht, Heidelberglaan 2, 3584 CS, Utrecht, The Netherlands; 2 Institute of Planetary Research, German Aerospace Center, Rutherfordstrasse 2, Berlin DE-12489, Germany; 3 Department of Physical Sciences, Open University, Walton Hall, Milton Keynes MK7 6AA, UK; 4 Department of Earth Sciences, University of Gothenburg, Gothenburg SE-405 30, Sweden

## Abstract

Liquid water is currently extremely rare on Mars, but was more abundant during periods of high obliquity in the last few millions of years. This is testified by the widespread occurrence of mid-latitude gullies: small catchment-fan systems. However, there are no direct estimates of the amount and frequency of liquid water generation during these periods. Here we determine debris-flow size, frequency and associated water volumes in Istok crater, and show that debris flows occurred at Earth-like frequencies during high-obliquity periods in the last million years on Mars. Results further imply that local accumulations of snow/ice within gullies were much more voluminous than currently predicted; melting must have yielded centimetres of liquid water in catchments; and recent aqueous activity in some mid-latitude craters was much more frequent than previously anticipated.

## Abstract (web summary)

It is thought that water flowed on the surface of Mars in the geological past during periods of high orbital obliquity. Here, the authors assess how much liquid water was present and suggest that debris flows occurred at Earth-like frequencies during high-obliquity periods in the past million years.

## Graphical abstract

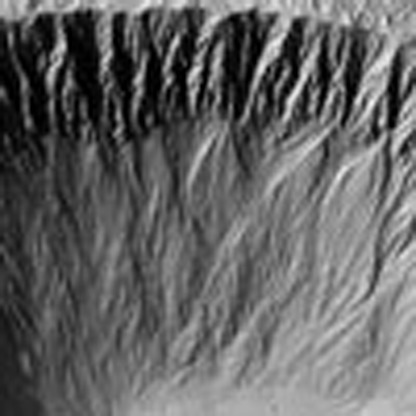

At present Mars is very cold and dry and its thin atmosphere makes liquid water at its surface exceptionally rare^1^,^2^. However, climatic conditions differed during periods of high-orbital obliquity in the last few millions of years^3^,^4^,^5^,^6^. In these periods liquid water was probably more abundant, as testified by the presence of numerous mid-latitude gullies, which are small catchment-fan systems^7^,^8^,^9^,^10^,^11^,^12^ (Fig. 1). During high-obliquity periods on Mars (>30°), increased polar summer insolation enhances polar ice sublimation, which increases atmospheric water content and amplifies circulation, leading to a more intense water cycle^4^,^5^. Precipitation of snow and ice is thought to become widespread in the mid-latitudes (from the poles to ∼30° N and S), and in the high mountains in lower-latitude regions^4^,^5^,^6^, leading to extensive glaciation^4^,^5^. Snow/ice probably melted during high-obliquity periods in favourable locations, forming thousands of gullies in the mid-latitudes^7^,^8^,^9^. Evidence of water-free sediment flows^13^,^14^,^15^, debris flows^8^,^10^ and fluvial flows^9^,^11^ has been identified, which result in a large morphological diversity of gullies on Mars. The morphology and morphometry of many gullies imply that they are formed by liquid water^9^,^10^,^11^,^12^, whilst other gullies are morphologically active today, probably driven mainly by CO_2_ frost, suggesting a water-free continuing present-day activity^13^,^14^. Some aqueous gullies formed in the last few millions of years^10^,^16^,^17^. As such, they are the youngest record of liquid water and extensive aqueous activity on the surface of Mars, and therefore of critical importance in resolving the planet's recent hydrologic and climatic history. Obliquity on Mars has varied between 15° and 35° in the last 5 Myr, in cycles of approximately 120 Kyr^18^. The obliquity threshold for snow and ice transfer from the poles to lower latitudes is estimated at 30° 3, whereas the threshold for melting and associated morphological activity is probably higher but unknown^19^.

Key questions that remain unanswered are how much water could potentially melt during these high-obliquity periods? And how frequent was the aqueous activity within the gullies? Here, we address these questions by quantifying debris-flow size, frequency and associated liquid water content on Mars, in the very young Istok crater in Aonia Terra (Fig. 1) (formed 0.1–1 Myr ago; best-fit age: ∼0.19 Ma; 45.11° S; 274.2° E)^10^. These analyses show that local accumulations of snow/ice within gullies were in the order of centimetres to decimetres during periods of high obliquity in the last Myr. Melting of this snow/ice must have yielded centimetres of liquid water in the gully catchments to produce the observed debris-flow volumes. Moreover, debris flows were much more frequent than previously anticipated and occurred at Earth-like frequencies in Istok crater at high obliquity.

## Results

### Study crater

The pole-facing slope of Istok crater hosts a bajada, a series of coalescing fans, with abundant debris-flow deposits^10^, which are among the best preserved found on Mars to date. Although morphometric analyses suggest that many gullies are formed by debris flows^12^, evidence thereof is generally absent on gully-fan surfaces. This is probably caused by post-depositional reworking of gully-fan surfaces by weathering and erosion^20^ or emplacement of latitude-dependent mantle deposits^10^ (LDM; a smooth, metres-thick deposit comprising layers of dust and ice that extends from the poles to the mid-latitudes^21^,^22^). The unusually pristine debris-flow deposits in Istok crater therefore make it the best, and only, crater wherein detailed quantitative analyses using debris-flow volumes can be performed today.

In Istok crater, contribution of fluvial and water-free sediment flows to the fan surface morphology on the bajada appears to be very minor. We base this on the absence of bright and dark deposits and ‘fingering' depositional lobes, which are both associated with dry sediment flows on Mars^13^,^14^,^15^, the abundance of well-developed leveed channels that have to date not been observed in recent dry flows on Mars; the remarkably good morphological and textural agreement with terrestrial debris flows^10^; and the 8° to 20° depositional slopes, typical for non-cohesive terrestrial debris flows^10^. In contrast, the landforms on the crater slopes with non-polar azimuths appear unrelated to liquid water^10^. This landform disparity further supports debris-flow formation by insolation-driven melting of snow/ice, because melting is hypothesized to mainly occur on pole-facing slopes at high obliquity in the mid-latitudes^8^,^9^. In contrast to many other gullies on Mars, the source of water seems to be unrelated to the LDM. This is testified by the presence of highly brecciated alcoves hosting many boulders, solely exposing bedrock and the abundance of metre-sized boulders on the depositional fans. The absence of LDM in the gullies is further supported by the lack of landforms associated with the LDM, such as polygonally patterned ground and moraine-like ridges^10^. The absence of LDM suggests that the debris flows in Istok crater formed from top-down melting of relatively pure (that is, little dust) snow packs^10^, and therefore inferences from debris-flow volumes, directly relate back to snowfall amounts and climate.

### Debris-flow volume and frequency

Debris flows are high-concentration mixtures of solid particles and water that move as a single-phase high-density flow. Non-cohesive debris flows contain ∼20–60% water by volume^23^,^24^,^25^. They form deposits with paired levees and distinct depositional lobes that often incorporate large boulders. We use the distinct morphology of these deposits to estimate individual debris-flow volumes from a High-Resolution Imaging Science Experiment (HiRISE) Digital Elevation Model (DEM) with a sampling distance of 1 m. Estimated individual debris-flow volumes roughly range from 400 to 5,100 m^3^ (Fig. 2a–c; Table 1) and are similar to those in unconfined terrestrial debris-flow systems^26^,^27^,^28^ (Fig. 3).

We estimated the total number of debris flows by comparing the volume of a single, modal-sized, debris flow to the total volume of sediment eroded from the catchments. Here, an alcove is defined as a single source area divided by ridges, and a catchment as a set of alcoves that together feed a similar part of the bajada (Supplementary Fig. 1). In total, around 28,000 modal-sized debris flows were needed to form the entire bajada and ∼1,900 debris flows originated from each catchment (Supplementary Table 1). From this we calculated the cumulative time above a specific obliquity threshold for melting and then determined the debris-flow frequency within the gullies, expressed as their return period^26^. Debris-flow return periods ranged between 4 and 15 years on the bajada, and 64–221 years in the catchments for a conservative obliquity threshold for melting of 30°. A melting threshold of 35° 19 implies return periods of 0.2–0.8 year for the bajada and 3–12 years for the catchments (Fig. 2d,e; Supplementary Table 1).

### Liquid water volumes

Using the known range of water concentrations of terrestrial debris flows in combination with the measured debris-flow volumes in Istok crater, we can make an estimation of the amount of liquid water required for each flow. The associated liquid water volume yields a minimum estimate of snow/ice deposition and subsequent melting within the alcoves. Between 3 and 9 mm of liquid water uniformly spread over an average-sized alcove is required for the formation of modal-sized debris flows, and 16–50 mm of liquid water is required for the formation of large, 95 percentile-sized debris flows (Table 1). The actual thickness of the snow/ice layer must have been much larger because of the porosity of the snowpack, potential sublimation and evaporation losses, and the fact that uniform melting over an entire alcove will generally not occur^19^. On the other hand, snowdrift might have led to larger accumulations of snow in the alcoves than was originally emplaced^29^. Potential infiltration losses are likely negligible on Mars where a frozen permafrost layer acts as an aquiclude^11^. Therefore, we estimate that centimetres to decimetres of snow must have accumulated in the alcoves to form the observed debris-flow deposits.

## Discussion

The surprisingly short debris-flow return periods at high orbital obliquity in Istok crater are very similar to those in various environments on Earth^26^,^27^,^28^ (Fig. 3). Moreover, they are similar or even shorter than in terrestrial environments that are climatologically comparable to the dry and cold polar desert of Mars. In one of the driest regions on Earth, the hyperarid Atacama Desert, debris-flow return periods along the coast of northern Chile and southern Peru range between 40 and 2,400 years^30^ and are probably higher further inland where precipitation is even less frequent. In the periglacial, polar semi-desert of Svalbard, which displays many periglacial landforms similar to Mars^31^, debris-flow return periods ranging between 80 and 500 years were found^32^. These results suggest that pole-facing mid-latitude crater walls on Mars, at least in Istok crater, were extremely active environments with Earth-like debris-flow activity during high-obliquity periods in the last few million years. Moreover, the debris-flow return periods during high obliquity are similar to present-day return periods of dry, CO_2_-aided, sediment flows in some of the currently most active gullies on Mars^14^. The debris-flow return periods in Istok crater imply that these gullies were among the most active aqueous gullies on Mars in the last Myr. They are the first estimated return periods of aqueous activity for Martian gullies. Yet, it is not unlikely that similar activity has occurred on other sites in the past.

Generally, inferences drawn from Global Climate Models (GCMs) suggest that annual atmospheric precipitation of snow/ice at 35° obliquity does not exceed a spatially averaged 10 mm per year^6^,^33^, and precipitation is even less at lower obliquity. However, recently Madeleine *et al.*^5^ incorporated the effect of radiatively active water-ice clouds in their Global Climate Model, resulting in annual snow/ice accumulations of ∼10 cm in the mid-latitudes at 35° obliquity, which corresponds well with the snow accumulation inferred from debris-flow volumes at Istok crater. Yet, although snowdrift causes larger accumulations of snow within the alcoves and snow might accumulate over multiple years, most snow is thought to sublimate^6^,^19^. Moreover, Williams *et al.*^19^ estimate that only a very small amount, in the order of 1 mm per year, of liquid water was produced by melting on pole-facing mid-latitude crater walls out of a 5-cm-thick snowpack at high obliquity. Kite *et al.*^34^ estimate that melting over a Mars-year produces 9 mm of liquid water in total. Clearly, these models do not explain the amounts of liquid water needed for the formation of the debris flows in Istok crater. This implies that melting of snow/ice must locally have been much larger than currently predicted by most climate models, and supports the recent improvements to the climate models by Madeleine *et al.*^5^.

We conclude that debris flows occurred at Earth-like frequencies in Istok crater during high-obliquity periods in the last million years on Mars. Although this required much more atmospheric deposition and subsequent melting than is currently predicted by climate models, these findings fit well into the emerging view of a much more dynamic recent and present Mars than anticipated only a few years ago^1^,^14^. Mars was long presumed to be a hyperarid environment dominated by wind since the onset of the Amazonian period, 3 Gyr ago. However, pristine debris-flow deposits in Istok crater provide compelling evidence for very active aqueous environments on pole-facing slopes in the mid-latitudes during high obliquity in the last million years. The surprisingly large amount of liquid water on these slopes means we should revise our understanding of Mars' recent climate, but also points to more habitable recent environments than previously predicted.

## Methods

### Production of the digital elevation model

The DEM used for the extraction of alcove and debris-flow volumes was constructed using the methods described by Kirk *et al.*^35^, from HiRISE stereo images PSP_006837_1345 and PSP_007127_1345. The ground sampling distance of the DEM is 1 m. The vertical precision of the DEM can be estimated based on viewing geometry and pixel scale. The stereo convergence angle of the HiRISE images is 20.1°, the largest spatial resolution of the two images is 0.258 m, and assuming 1/5 pixel correlations yields a vertical precision of 0.258/5/tan(20.1)=0.13 m^35^.

### Extraction of alcove and debris-flow volumes

The volume of material eroded from the alcoves was determined from the DEM. Following Conway and Balme^22^ we assume that the top of the alcove crests represent the initial pre-gully surface. In reality, alcove crests will also be eroded, and thus our assumption yields a lower bound estimate. The eroded volume was derived by subtracting the original from the pre-gully surface. Error propagation calculations by Conway and Balme^22^ suggest that such volume estimates are accurate within 15%. Because a bajada is composed of a series of coalescing fans, it is impossible to directly determine total bajada volume given the uncertainty of the pre-gully crater profile. However, as (1) the material eroded from the alcoves consists of pure bedrock, unrelated to the LDM, and (2) the composition and rheology of debris flows prevent significant escape of material after deposition, the gullies are probably closed systems wherein the amount of sediment eroded from the alcoves is approximately equal to the amount of material deposited on the bajada, as quantitatively demonstrated by Conway and Balme^22^. We therefore approximate the total bajada volume by calculating the total amount of material eroded from the alcoves. As such, we neglect the potential input of material by rockfalls to the bajada. However, the abundance of debris-flow deposits on the bajada suggests that the volume transfer by rockfalls is minor compared with the transfer by debris flows.

Individual debris-flow volumes were determined from the orthorectified image and from the DEM. We measured width, length and height of 144 clearly resolvable lobes and width and height of 70 levees (Supplementary Fig. 1; Supplementary Tables 3 and 4), which we subsequently combined into debris-flow volume based on percentiles (Fig. 2a–c). Width and length were measured using the orthorectified image, and height using the DEM. We combined percentiles of lobe and levee size to obtain debris-flow volumes, because it was impossible to directly determine debris-flow volumes, as the debris flows on the bajada are strongly amalgamated. We avoided measurement of lobes that were largely buried by subsequent debris flows to prevent an under-prediction of lobe volume (see Supplementary Fig. 2 for an example of the delineation of lobes). It is impossible to determine whether the debris flows in Istok crater formed one or multiple lobes, based on remote sensing data only. However, on Earth it is much more common for debris flows to form one lobe rather than multiple lobes^27^,^32^,^36^ and there is no model suggesting that forking should occur more frequently under Martian conditions. Therefore, we assume that each debris flow formed one depositional lobe. This might underestimate the volume of very large debris flows, which can bifurcate and form multiple lobes, but is probably a good representation of the modal-sized debris flow and therefore debris-flow return periods. To account for errors associated with the estimation of the debris-flow cross-section, levee and lobe volumes were calculated by assuming a triangular (minimum estimate), rectangular (maximum estimate) and a trapezoidal cross-section (intermediate estimate) (Supplementary Fig. 3). Total levee volume for the modal-sized debris flows was calculated by assuming paired levees of half the bajada length (900 m; 800–1,100 m range), where we used the mean, minimum and maximum length for the calculation of the intermediate, minimum and maximum volume estimates, respectively. This is a rough estimation, but as accurate direct measurement of modal debris-flow length is very ambiguous half the bajada length was chosen as a parsimonious approximation. This assumption will lead to an underestimation of the volume for relatively long debris flows and an overestimation for relatively short debris flows, but is a good approximation of the modal-sized debris flow and therefore the return periods.

### Estimation of debris-flow return periods

**Figure 1:**
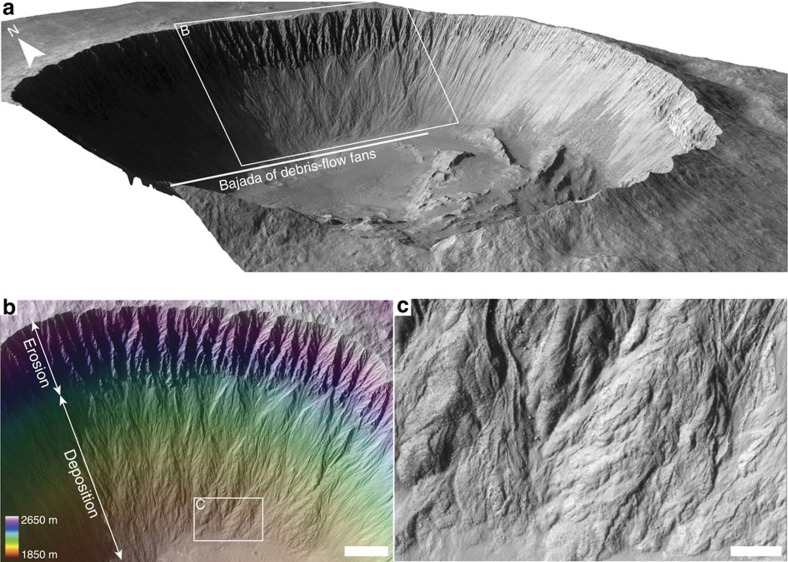
Istok crater. (**a**) Bajada of remarkably pristine debris-flow fans on the pole-facing slope (45.11° S; 274.2° E). The bajada length is ∼4 km. (**b**) Eroding alcoves supply sediments to the downslope bajada of fans. Scale bar, 250 m wide. (**c**) The fans are composed of debris-flow deposits, as testified by the widespread occurrence of paired levees, distinct depositional lobes and embedded boulders^10^. Scale bar, 50 m wide.

**Figure 2:**
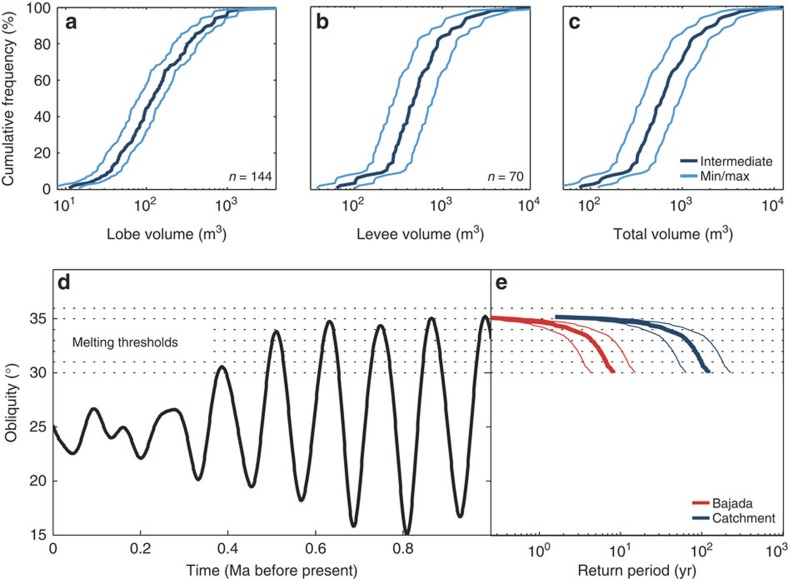
Debris-flow return periods and size in Istok crater. (**a**) Cumulative frequency distribution of lobe volume. The minimum, maximum and intermediate estimates are based on a triangular, rectangular and trapezoidal-shaped lobe model, respectively (Supplementary Fig. 2). (**b**) Cumulative frequency distribution of levee volume, estimated by assuming paired levees of half the bajada length (900 m; 800–1,100 m range). The minimum estimate is based on triangular-shaped paired levees of 400 m long, the maximum estimate on rectangular-shaped paired levees of 550 m long, and the intermediate estimate on trapezoidal-shaped levees of 450 m long. (**c**) Cumulative frequency distribution of total debris-flow volume (lobe and levee volume combined). (**d**) Obliquity in the last Myr on Mars^18^, and potential thresholds for melting on mid-latitude pole-facing crater walls. (**e**) Debris-flow return periods on the bajada and per catchment. The intermediate estimate (thick line) is calculated from the intermediate-estimate debris-flow size and best-estimate catchment size. The minimum and maximum estimates are calculated from the largest debris-flow size and smallest catchment volume and the smallest debris-flow size and maximum catchment volume, respectively. See Supplementary Tables 1–5 for raw data.

The debris-flow return periods were calculated by dividing the cumulative time above an obliquity threshold (Fig. 2d)^18^,^19^ since the formation of Istok crater, by an estimate of the total number of debris flows on the bajada and per catchment. We conservatively estimate return periods by using the maximum age of Istok crater (formed 1 Ma)^10^. The number of debris flows was calculated by dividing the volume of sediment eroded from the alcoves by the volume of the modal-sized debris flow that formed the bajada. As we can only observe, and therefore measure, the volume of surficial debris flows, we assume that volumes remained quasi-static over time. Approximately 45% of the pole-facing crater wall hosting the fans is covered in shadows, and hence debris flows were not accurately discernible (Fig. 1). Therefore, we calculated total volume on the bajada by extrapolating the volume on the sun-illuminated part to the shadowed part by assuming similar geometry. Return periods were calculated for the entire bajada and per average-sized catchment and for a range of obliquities exceeding 30°.

### Robustness of results

Our calculations are based on a number of assumptions. However, this is inevitable for the current analysis that can only be based on remote sensing data. For this reason we conservatively estimated possible errors and propagated these through our calculations. Moreover, we conservatively calculated alcove volumes and used the maximum estimated age of the host crater as an overestimate of the gully-system age. Even then, the uncertainty in age range (up to a factor of 5: 0.2–1.0 Myr) is larger than the error associated with volume calculations. For example, if we adopt the extreme scenario that every debris flow formed two lobes and that we underestimated lobe volume by a factor of 2 (for example, by underestimating runout length or missing part of the lobe volume due to partial burial), this results in an error of a factor of 4 in lobe volumes and associated return periods and liquid water volumes. As such, this error is smaller than the error range associated with crater age uncertainty.

**Figure 3:**
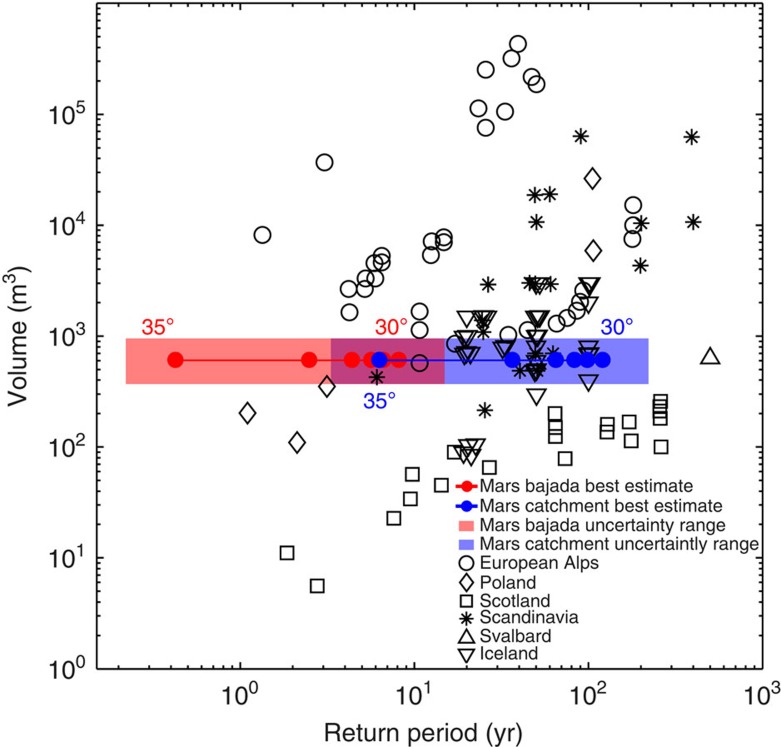
Debris-flow volumes and return periods in Istok crater and examples from Earth. The return periods on Mars are clearly within the range of return periods observed in temperate to polar regions on Earth regardless of the uncertainty in debris-flow volume, return periods per bajada or individual catchment and obliquity thresholds for melting between 30° and 35°. Data from the European Alps, Poland, Scotland, Scandinavia and Svalbard are from Van Steijn^26^ and references therein, additional data for the Alps from Helsen *et al.*^27^, and Icelandic data from Decaulne and Sæmundsson^28^.

Second, our conclusions on debris-flow return periods are largely insensitive to the assumptions. If we adopt an extreme scenario where we overestimate or underestimate debris-flow volume by a factor of 4, the number of debris flows that formed the bajada, and the return periods also change by a factor of 4. However, compared with the large range of debris-flow return periods observed on Earth this is not significantly different (Fig. 3). Neither does a factor 4 difference in liquid water and snow accumulation volumes change the result that centimetres to decimetres of snow were required for the formation of debris flows. Therefore, the conclusions are robust.

## Additional information

**How to cite this article:** de Haas, T. *et al.* Earth-like aqueous debris-flow activity on Mars at high orbital obliquity in the last million years. *Nat. Commun.* 6:7543 doi: 10.1038/ncomms8543 (2015).

## Supplementary Material

Supplementary Information Supplementary Figures 1-3, Supplementary Tables 1-5 and Supplementary References

## Figures and Tables

**Table 1:** Minimum amounts of liquid water required for the generation of debris flows.

**Debris-flow size**	**Debris-flow volume (m**^3^)	**Water:sediment ratio 0.2**		**Water:sediment ratio 0.6**	
		**Water volume (m**^3^)	**Water in alcove (mm)**	**Water volume (m**^3^)	**Water in alcove (mm)**
Modal	605 (368–950)	121 (74–190)	3.0 (1.8–4.7)	363 (221–570)	9.0 (5.5–14.1)
95% largest	3,307 (2,031–5,101)	661 (406–1,020)	16.4 (10.0–25.2)	1,984 (1,219–3,061)	49.1 (30.1–75.7)

Error margins expressed as minimum and maximum values within brackets (see Fig. 2a–c and Supplementary Tables 3 and 4 for raw debris-flow volume data, and Supplementary Table 2 for raw alcove volume data).
