# Supplementary material for: Earth-like aqueous debris-flow activity on Mars at high orbital obliquity in the last million years
Source: Nat Commun. 2015 Jun 23;6:7543. doi: 10.1038/ncomms8543 (PMC4557294; doi:10.1038/ncomms8543)
Supplement: Supplementary Information — Supplementary Figures 1-3, Supplementary Tables 1-5 and Supplementary References [file ncomms8543-s1.pdf]

Supplementary Information

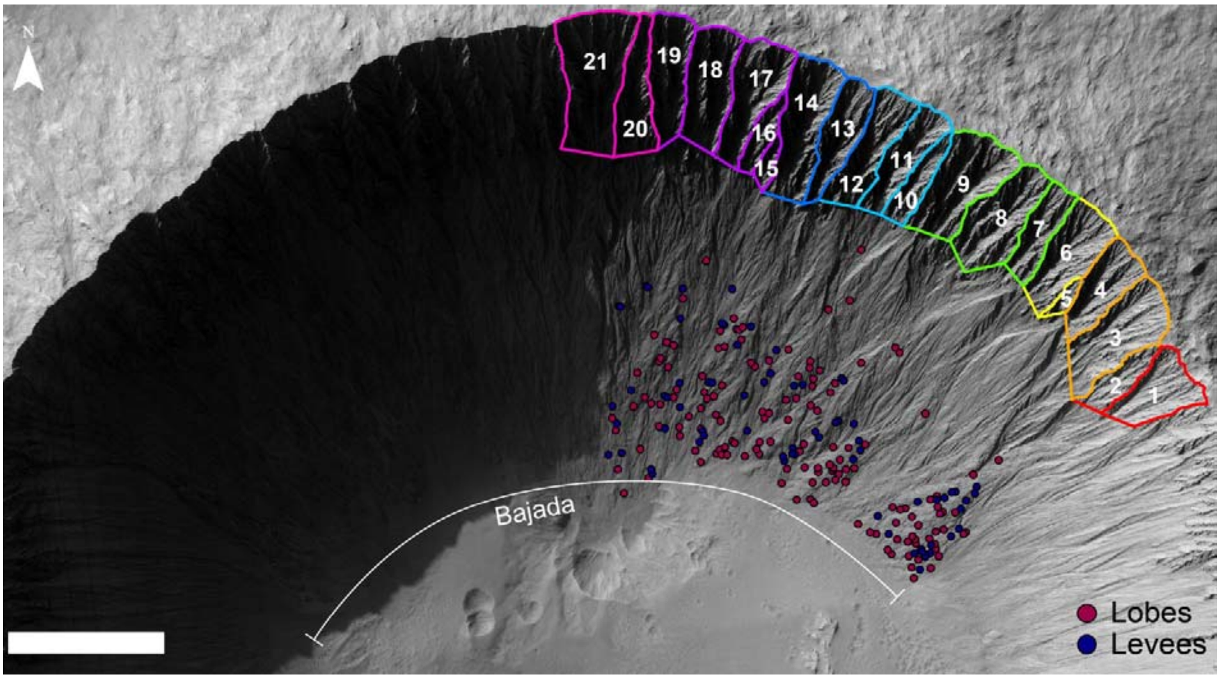

Supplementary Figure 1. Measurement locations. The numbered alcoves denote all the alcoves that are separated by high crests. Coloured outlines denote sets of alcoves that together form catchments that feed similar parts of the bajada. Alcove numbers correspond to numbers in Supplementary Table 2. The scale bar is 500 m wide.

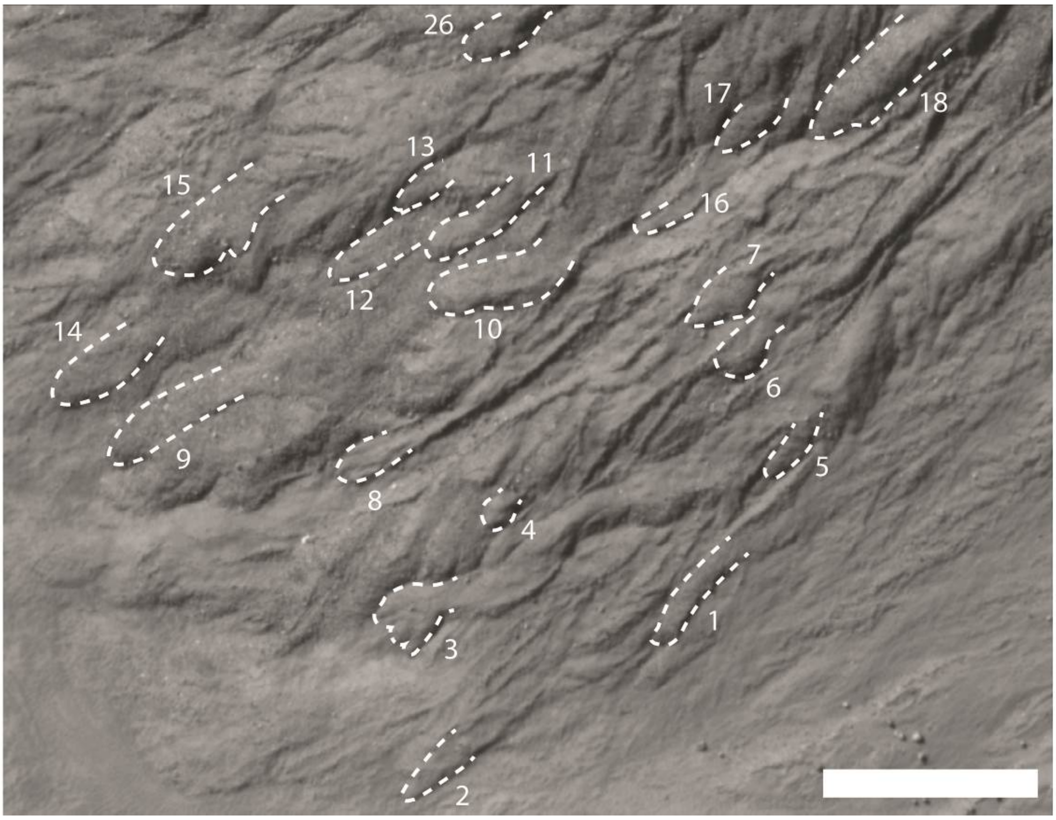

Supplementary Figure 2. Delineation of debris-flow lobes. Where possible, lobes are delineated up to the transition to leveed channel; otherwise the visible part of the lobe was delineated. Note that in the latter case parts of the lobe were not measured, which might have caused an underestimation of lobe volume. To minimize this error we avoided lobes for which large parts were clearly or likely buried by subsequent deposits. Numbers correspond to numbers in Supplementary Table 3. Scale bar is 50 m wide.

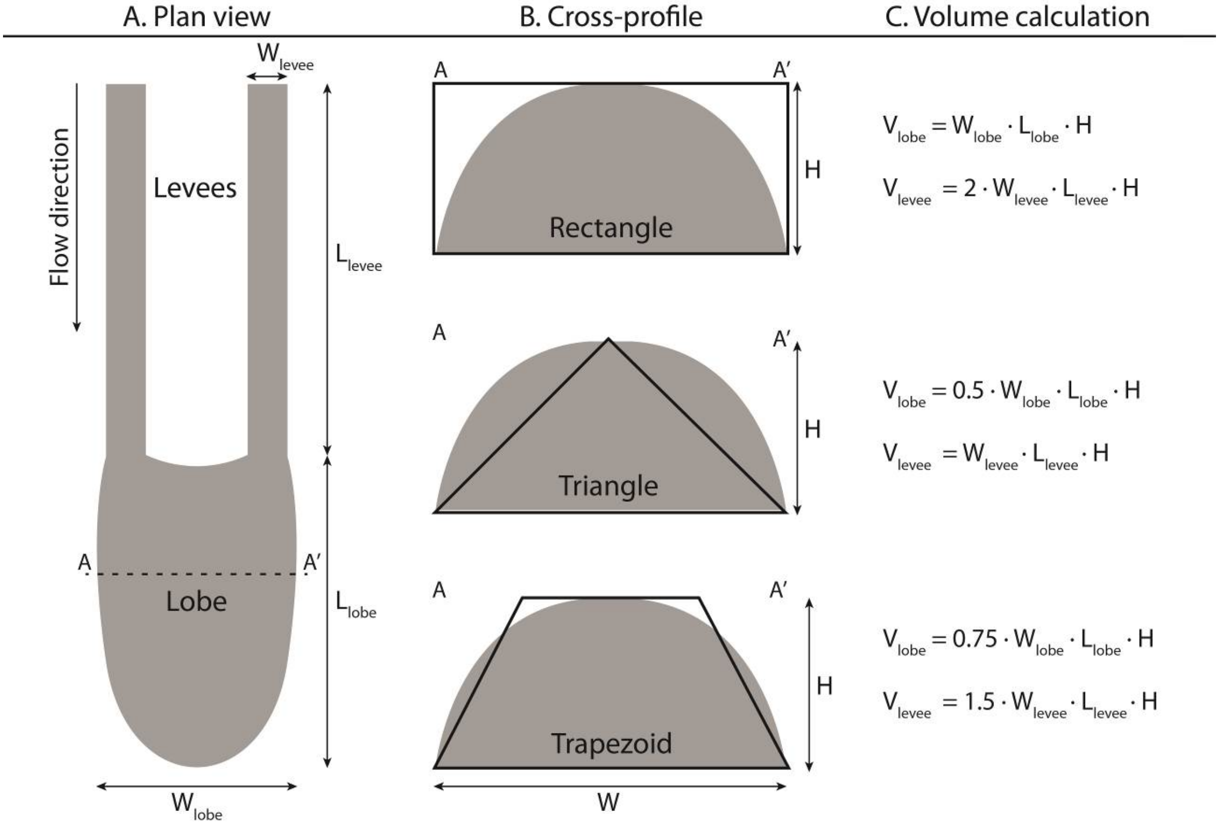

Supplementary Figure 3. Debris-flow volume calculations. (A) Plan view of a typical debris flow, wherein the bulk of sediment is stored in paired levees and a depositional lobe. (B) Potential debris-flow lobe and levee cross-profile approximation models. A rectangular model likely overestimates the volume and a triangular model probably underestimates the volume. As such, we used the trapezoidal model as an intermediate estimate, whereas the triangular and rectangular models are used as minimum and maximum estimates, respectively. (C) Equations used to calculate total lobe and levee volume for each model.

Supplementary Table 1. Debris-flow return periods on the bajada and averaged per catchment for a range of obliquity values between 30° and 36°.

	**Bajada**			**Catchment**		
	Intermediate	Max.	Min.	Intermediate	Max.	Min.
Eroded volume (m^3)	$1.72x10^7$	$1.46x10^7$	$1.97x10^7$	$1.16x10^6$	$0.98x10^6$	$1.33x10^6$
Debris flow volume (m^3)	605	368	950	605	368	950
Number of debris flows	28348	15346	53665	1911	1035	3618
Return period (yr):						
30° obliquity	8.08	14.92	4.27	119.83	221.34	63.30
31° obliquity	6.60	12.19	3.48	97.85	180.75	51.69
32° obliquity	5.61	10.36	2.96	83.20	153.68	43.95
33° obliquity	4.37	8.08	2.31	64.88	119.85	34.27
34° obliquity	2.47	4.56	1.30	36.63	67.66	19.35
35° obliquity	0.42	0.78	0.22	6.28	11.60	3.32
36° obliquity	0.00	0.00	0.00	0.00	0.00	0.00

Supplementary Table 2. Alcove area and eroded volume. The best estimate is the measured value, whereas the minimum and maximum estimates are derived using the method uncertainty of 15%[1]. Alcove numbers correspond to numbers in Supplementary Fig. 1.

Alcove	Area (m2)	Eroded volume (m^3) best estimate	Eroded volume (m^3) minimum estimate	Eroded volume (m^3) maximum estimate
1	40208	333945	283854	384037
2	20367	107370	91264	123475
3	61645	672103	571287	772918
4	38729	439377	373470	505284
5	5609	27801	23631	31971
6	48781	616602	524112	709092
7	28106	202848	172421	233275
8	56518	634682	539480	729884
9	62159	824061	700452	947670
10	20340	141539	120308	162770
11	35859	390305	331760	448851
12	42884	342386	291028	393744
13	36822	393911	334825	452998
14	61671	960823	816700	1104947
15	7418	51777	44011	59544
16	14630	96387	81929	110845
17	48835	648526	551247	745805
18	51409	492512	418635	566389
19	46141	511917	435129	588704
20	39449	417962	355267	480656
21	81439	947756	805593	1089920
Average	40429	440695	374591	506799
Maximum	81439	960823	816700	1104947
Minimum	5609	27801	23631	31971

Supplementary Table 3. Debris-flow lobe size. See Supplementary Fig. 3 for the volume calculation methods.

Lobe	width (m)	length (m)	height (m)	volume rectangle (m^3)	volume triangle (m^3)	volume trapezoid (m^3)
1	4.6	23.0	0.4	43	22	33
2	5.3	10.7	0.5	28	14	21
3	13.0	12.0	0.7	115	57	86
4	6.9	9.4	0.8	53	27	40
5	5.4	15.7	1.2	104	52	78
6	7.9	16.6	1.7	220	110	165
7	8.7	18.2	1.3	200	100	150
8	7.2	17.6	0.7	90	45	68
9	9.5	31.0	2.3	672	336	504
10	9.4	23.4	0.9	203	102	152
11	6.4	21.0	0.7	101	50	76
12	6.2	24.0	1.0	148	74	111
13	5.8	18.0	1.2	123	61	92
14	9.5	27.0	1.6	401	201	301
15	12.6	36.0	2.0	908	454	681
16	4.1	14.4	0.5	30	15	22
17	7.0	15.7	1.4	150	75	112
18	9.8	31.2	1.8	542	271	407
19	4.7	12.1	1.6	93	46	70
20	4.3	10.6	0.4	20	10	15
21	4.9	11.7	0.9	53	26	40
22	9.6	21.0	0.7	141	70	106
23	9.6	20.6	0.8	148	74	111
24	7.4	11.9	0.9	77	38	58
25	7.8	17.8	1.2	166	83	124
26	7.1	16.5	1.9	217	108	163
27	6.9	9.4	1.7	111	56	83
28	4.9	10.3	1.1	57	28	43
29	5.0	7.9	1.5	61	30	46
30	12.6	22.2	0.8	214	107	161
31	8.0	37.0	0.7	194	97	145
32	5.9	12.5	0.9	63	32	47
33	4.8	16.0	1.7	128	64	96
34	4.3	15.7	1.3	88	44	66
35	4.6	15.3	0.9	63	31	47
36	4.7	7.9	1.0	36	18	27
37	5.9	19.2	2.3	257	129	193
38	5.3	13.7	0.8	58	29	44
39	12.2	27.6	1.0	342	171	256
40	13.6	22.8	1.1	349	174	262
41	8.3	16.5	1.0	131	65	98
42	6.6	14.9	1.7	167	83	125
43	14.6	40.0	3.1	1783	892	1337
44	8.3	22.7	2.2	407	204	306
45	5.7	10.3	0.3	17	8	13
46	9.0	22.0	2.0	391	196	294
47	5.3	12.5	0.9	60	30	45
48	7.8	13.3	1.8	184	92	138
49	5.7	14.1	0.9	73	36	54
50	5.4	11.7	1.5	97	48	73
51	5.9	9.7	1.1	61	31	46
52	8.8	18.8	0.8	139	70	104

53	5.7	13.3	1.4	109	54	82
54	7.0	11.2	2.0	158	79	118
55	13.0	50.0	2.0	1287	644	966
56	8.2	11.0	0.9	85	43	64
57	8.4	38.0	1.1	349	174	261
58	8.5	24.0	2.0	414	207	310
59	10.7	19.6	0.5	110	55	83
60	6.0	13.3	0.6	49	25	37
61	30.1	65.4	4.7	9202	4601	6901
62	18.6	80.0	0.9	1312	656	984
63	12.6	26.0	2.3	738	369	554
64	8.1	27.8	4.1	934	467	701
65	10.3	22.3	0.6	144	72	108
66	7.8	29.6	1.8	423	211	317
67	9.3	12.4	0.8	97	48	73
68	5.5	21.4	1.1	127	63	95
69	5.3	8.4	0.4	16	8	12
70	6.8	18.2	0.6	72	36	54
71	5.9	15.1	0.7	67	33	50
72	3.1	9.9	0.5	15	7	11
73	19.1	37.4	1.9	1393	696	1044
74	8.9	20.0	1.3	226	113	169
75	6.9	13.1	0.5	47	23	35
76	11.2	19.8	1.0	213	106	159
77	12.6	23.8	0.8	228	114	171
78	11.8	12.8	2.9	443	222	333
79	7.9	21.2	0.8	130	65	97
80	8.5	21.7	2.9	530	265	398
81	5.9	15.3	1.0	93	46	69
82	12.4	31.0	2.4	912	456	684
83	19.8	53.0	3.6	3745	1872	2809
84	6.1	13.4	1.1	90	45	67
85	8.8	41.3	1.6	595	298	446
86	7.6	17.7	1.2	165	82	124
87	4.7	14.0	0.4	25	13	19
88	7.2	15.5	0.7	76	38	57
89	7.6	25.1	1.7	316	158	237
90	5.3	13.9	1.1	81	40	60
91	7.9	21.8	1.4	246	123	185
92	5.6	12.3	1.9	128	64	96
93	5.1	13.1	1.0	70	35	52
94	6.9	15.3	3.5	373	186	280
95	17.7	45.3	1.3	1038	519	779
96	8.5	33.9	0.9	262	131	196
97	6.7	21.7	1.2	174	87	131
98	7.2	44.7	0.9	294	147	220
99	6.7	19.8	1.7	224	112	168
100	9.7	46.2	3.0	1357	678	1018
101	9.1	18.4	0.7	125	63	94
102	6.0	14.0	0.5	40	20	30
103	6.6	13.7	1.3	118	59	89
104	6.3	12.1	0.8	59	29	44
105	7.7	17.1	0.8	101	51	76
106	9.8	28.7	1.7	483	241	362
107	6.6	8.2	0.7	35	18	27
108	7.1	17.4	0.4	50	25	37
109	7.3	24.1	1.2	205	102	154

110	6.4	14.8	1.3	123	61	92
111	8.4	32.5	1.3	352	176	264
112	9.6	44.1	2.0	855	427	641
113	6.9	24.5	2.7	457	228	343
114	8.9	32.6	1.5	421	211	316
115	11.7	34.6	1.3	507	254	380
116	10.9	30.2	2.0	666	333	499
117	6.5	13.5	1.3	111	55	83
118	12.9	29.2	1.3	480	240	360
119	6.8	10.4	0.5	32	16	24
120	5.2	12.8	1.0	64	32	48
121	6.1	15.4	1.8	170	85	128
122	8.0	18.8	0.6	96	48	72
123	14.6	24.9	0.7	250	125	187
124	6.5	20.6	0.9	124	62	93
125	7.6	18.6	1.4	193	97	145
126	12.1	23.7	1.1	326	163	244
127	6.4	14.4	0.5	50	25	38
128	16.2	20.6	2.1	701	351	526
129	9.3	14.3	3.0	402	201	302
130	7.4	11.2	0.6	50	25	38
131	6.5	18.6	1.6	191	95	143
132	11.4	30.5	1.1	398	199	298
133	8.8	31.5	0.7	182	91	136
134	7.5	16.7	1.3	159	80	120
135	12.5	31.0	1.6	629	315	472
136	10.0	25.6	0.8	212	106	159
137	6.0	10.0	0.7	43	22	32
138	10.3	33.2	2.7	931	466	698
139	9.0	11.4	1.2	123	62	92
140	11.3	25.7	4.6	1327	664	995
141	11.1	45.7	1.7	864	432	648
142	8.9	22.3	1.1	221	110	166
143	9.8	24.3	1.3	304	152	228
144	8.1	21.4	3.0	526	263	395
Median	7.7	18.6	1.2	162	81	122
Maximum	30.1	80.0	4.7	9202	4601	6901
Minimum	3.1	7.9	0.3	15	7	11

Supplementary Table 4. Debris-flow levee size per meter. See Supplementary Fig. 3 for the volume calculation methods.

Levee	width (m)	height (m)	volume rectangle (m^3 m^{-1})	volume triangle (m^3 m^{-1})	volume trapezoid (m^3 m^{-1})
1	1.24	1.06	1.31	0.65	0.98
2	1.24	0.83	1.03	0.51	0.77
3	1.08	0.33	0.35	0.18	0.27
4	1.44	0.41	0.59	0.29	0.44
5	0.82	0.18	0.15	0.08	0.11
6	1.43	0.31	0.44	0.22	0.33
7	1.55	0.27	0.41	0.21	0.31
8	1.99	0.35	0.70	0.35	0.52
9	1.71	0.44	0.76	0.38	0.57
10	1.86	0.60	1.12	0.56	0.84
11	1.35	0.87	1.17	0.58	0.88
12	1.20	0.37	0.44	0.22	0.33
13	1.00	0.49	0.49	0.24	0.37
14	1.26	0.43	0.54	0.27	0.40
15	1.12	0.47	0.53	0.26	0.39
16	1.00	0.16	0.16	0.08	0.12
17	1.18	0.29	0.35	0.17	0.26
18	1.34	0.61	0.82	0.41	0.61
19	1.60	0.26	0.41	0.20	0.31
20	0.88	0.11	0.09	0.05	0.07
21	3.80	0.23	0.88	0.44	0.66
22	1.30	0.48	0.62	0.31	0.46
23	1.71	0.44	0.75	0.38	0.56
24	3.90	1.40	5.48	2.74	4.11
25	2.00	0.56	1.11	0.56	0.83
26	1.30	0.36	0.47	0.23	0.35
27	4.10	1.10	4.51	2.26	3.38
28	2.35	0.55	1.29	0.65	0.97
29	2.60	0.57	1.47	0.74	1.11
30	2.10	1.21	2.55	1.27	1.91
31	1.40	0.11	0.15	0.08	0.11
32	2.00	0.57	1.15	0.57	0.86
33	1.60	1.11	1.78	0.89	1.33
34	2.00	0.35	0.70	0.35	0.53
35	1.36	0.30	0.41	0.21	0.31
36	1.30	0.48	0.63	0.31	0.47
37	2.10	0.29	0.61	0.30	0.46
38	1.57	0.78	1.23	0.61	0.92
39	1.54	0.35	0.54	0.27	0.40
40	2.51	0.41	1.03	0.52	0.77
41	2.85	0.67	1.91	0.95	1.43
42	2.99	0.17	0.50	0.25	0.37
43	2.38	0.33	0.80	0.40	0.60
44	2.18	0.05	0.10	0.05	0.08
45	2.30	0.37	0.86	0.43	0.65
46	1.90	0.25	0.48	0.24	0.36
47	1.60	0.26	0.41	0.20	0.31
48	2.00	0.29	0.59	0.29	0.44
49	1.78	0.12	0.21	0.10	0.15
50	1.31	0.38	0.50	0.25	0.37
51	1.47	0.47	0.69	0.34	0.52
52	1.90	0.23	0.43	0.22	0.32

53	1.92	0.68	1.31	0.66	0.98
54	2.87	0.64	1.83	0.91	1.37
55	2.46	0.98	2.41	1.21	1.81
56	2.40	0.39	0.94	0.47	0.70
57	1.70	0.37	0.62	0.31	0.47
58	1.37	0.22	0.30	0.15	0.23
59	1.29	0.60	0.77	0.39	0.58
60	2.68	0.99	2.65	1.32	1.99
61	3.19	1.08	3.46	1.73	2.59
62	1.55	0.69	1.08	0.54	0.81
63	2.67	0.30	0.81	0.41	0.61
64	1.67	0.22	0.36	0.18	0.27
65	6.30	2.78	17.49	8.75	13.12
66	1.26	0.58	0.73	0.37	0.55
67	1.18	0.51	0.60	0.30	0.45
68	2.70	0.50	1.36	0.68	1.02
69	3.30	0.84	2.77	1.38	2.07
70	2.90	0.28	0.80	0.40	0.60
Median	1.71	0.42	0.72	0.36	0.54
Maximum	6.30	2.78	17.49	8.75	13.12
Minimum	0.82	0.05	0.09	0.05	0.07

Supplementary Table 5. Time above obliquity threshold for melting[2].

Obliquity threshold	Time above threshold in last Ma (yr)
30°	229000
31°	187000
32°	159000
33°	124000
34°	70000
35°	12000
36°	0

Supplementary References

1. Conway, S. J., Balme, M. R., Decametre-thick remnant glacial ice deposits on Mars. G*eophys. Res. Lett*., **41(15)**, 5402-5409, (2014).
2. Laskar, J. *et al.*, Long term evolution and chaotic diffusion of the insolation quantities of Mars. *Icarus* **170**, 343-364 (2004).
